# TDP‐43 regulates the ubiquitination degradation of BMAL1, cellular glucoseutilization and energy absorption

**DOI:** 10.1002/alz.085715

**Published:** 2025-01-03

**Authors:** Jianlan Gu, Liti Zhang, Ruolan Yan, Dandan Chu, Fei Liu

**Affiliations:** ^1^ Nantong University, Nantong, NY China; ^2^ Department of Biochemistry and Molecular Biology, School of Medicine, Jiangsu China; ^3^ Nantong University, Nantong China; ^4^ New York State Institute for Basic Research in Developmental Disabilities, Staten Island, NY USA

## Abstract

**Background:**

Circadian rhythm disorder is not only a characteristic of neurodegenerative diseases but may participate in driving the pathological development in early stages of these diseases. Transactive response DNA‐binding protein of 43 kDa (TDP‐43) knockdown and its pathological aggregation are associated with severe neurodegenerative diseases such as amyotrophic lateral sclerosis.

**Methods:**

C57BL/6 mice were sleep deprived and sarcrificed at ZT0, ZT6, ZT12, and ZT18 and detected by Western blots. M17 cells infected with Lenti/TDP‐43_KO_ and then performed to RNA sequencing, and detected by qPCR to verify the RNA‐seq results. Mice were intracerebroventricular injected with AAV/shTDP‐43 and detected by Western blots and qPCR. HEK‐293T cells were transfected with TDP‐43, TDP‐43_KO_ or treated with MG132 or cycoheximide and performed to immunoprecipitation and detected by Western blots.

**Results:**

Herein we found that TDP‐43 expression exhibited rhythmic patterns and regulated the expression of multiple circadian genes such as BMAL1, CLOCK, CRY1, and PER2, particularly affecting the mRNA and protein levels of BMAL1; knockdown of TDP‐43 in mice brain also changed the autonomous circadian wheel behavior, cognitive and balance ability of the animal. Further, we discovered that TDP‐43 could at least regulate the expression and alternative splicing of USP13, thereby affecting the protein level of USP13 and the ubiquitin‐mediated degradation of BMAL1 and regulated the AMPK signaling pathway, resulted in changing the cellular glucose uptake and ATP production.

**Conclusion:**

Our findings would expand the understanding of the role of TDP‐43 dysfunction in circadian rhythm disruption in neurodegenerative diseases and provide new mechanistic evidences supporting the interaction between circadian rhythm disruption and neurodegeneration.

